# Epithelial–Mesenchymal Transition in Acute Leukemias

**DOI:** 10.3390/ijms25042173

**Published:** 2024-02-11

**Authors:** Lokman Varisli, Spiros Vlahopoulos

**Affiliations:** 1 Department of Molecular Biology and Genetics, Science Faculty, Dicle University, Diyarbakir 21280, Turkey; 2 First Department of Pediatrics, National and Kapodistrian University of Athens, Thivon & Levadeias 8, Goudi, 11527 Athens, Greece

**Keywords:** EMT, leukemia, neoplasia, cell adhesion, signaling

## Abstract

Epithelial–mesenchymal transition (EMT) is a metabolic process that confers phenotypic flexibility to cells and the ability to adapt to new functions. This transition is critical during embryogenesis and is required for the differentiation of many tissues and organs. EMT can also be induced in advanced-stage cancers, leading to further malignant behavior and chemotherapy resistance, resulting in an unfavorable prognosis for patients. Although EMT was long considered and studied only in solid tumors, it has been shown to be involved in the pathogenesis of hematological malignancies, including acute leukemias. Indeed, there is increasing evidence that EMT promotes the progression of acute leukemias, leading to the emergence of a more aggressive phenotype of the disease, and also causes chemotherapy resistance. The current literature suggests that the levels and activities of EMT inducers and markers can be used to predict prognosis, and that targeting EMT in addition to conventional therapies may increase treatment success in acute leukemias.

## 1. Introduction

Transitions between epithelial and mesenchymal phenotypes have been studied for decades, and characterized by their dynamic nature, which enables cells to adapt to new functions, according to the needs of the organism [1]. Epithelial cells that undergo epithelial–mesenchymal transition (EMT) lose their junctions and baso-apical polarity, reprogram their metabolism, and acquire a back-to-front polarity that confers them the ability to migrate and invade surrounding tissues. EMT enables the formation of the body plan and the differentiation of multiple tissues and organs. In a mature organism, EMT contributes to tissue repair. In pathology, EMT is involved in organ fibrosis and in cancer progression; in the latter, cells acquire developmental plasticity, migratory and invasive properties, and resistance to apoptosis, senescence, and destruction by the immune system [2].

EMT is activated by various dynamic stimuli from the local microenvironment, including growth factors and cytokines, hypoxia, and contact with the surrounding extracellular matrix (ECM); the mechanism entails specific switching of gene expression programs, which are initiated by transforming growth factor β (TGF-β) and bone morphogenetic protein (BMP), Wnt-β-catenin, Notch, Hedgehog, and receptor tyrosine kinases [3]. These switching programs in turn activate sequence-specific transcription factors (TFs) to turn on the expression of downstream genes [4]. These downstream genes are recognized as the hallmarks of EMT manifestation and encode structural proteins and cell adhesion molecules such as vimentin, N-cadherin, fibronectins, smooth muscle actin, as well as matrix metalloproteases [5]. It must be noted that TGF-β modulates inflammatory processes and has a pronounced impact on tissue homeostasis [6].

In the mammalian fetus, EMT stromal cells of the hepatic portal triads produce fibronectin, which is bound by late-stage erythroid cells to regulate their differentiation; EMT stromal cells transform the microenvironment to support the emergence, expansion, and maintenance of fetal hematopoietic development during the mid-gestational stage [7].

In the normal bone marrow (BM), hematopoietic stem and progenitor cell differentiation and hematopoietic lineage fidelity are controlled by TFs ZEB1 and ZEB2, which operate EMT signaling pathways [8,9]. Knocking out *Zeb2* in the BM promotes a phenotype with several features that resemble human myeloproliferative disorders, such as BM fibrosis, splenomegaly, and extramedullary hematopoiesis [10]. After colonizing the fetal liver, *Zeb2*-deficient hematopoietic stem/progenitor cells (HSPCs) exhibit altered adhesion and homing properties, and fail to reenter the blood circulation to colonize the BM cavity [8].

## 2. Variations of the EMT Theme in Cancer

In cancer, the activation of EMT switching mechanisms enables phenomena that are linked to de-differentiation and migration, which in combination with the reverse process from EMT, namely MET (mesenchymal to epithelial transition), facilitates metastasis [11,12,13,14,15,16]. What is important is that EMT-like phenomena confer a substantial degree of dynamic plasticity to cancer cell clones, which can function as cancer stem cells that have the properties of drug resistance and tumor initiation [17]. It must be noted that EMT and cancer stem cells (CSCs) do not represent a fixed state of phenotype, but reflect a dynamic flux of adaptive biological responses of malignant cells to drug treatment, oxidant stress, and metabolite alterations in their microenvironment; a characterized feature of CSCs, namely increased expression of aldehyde dehydrogenase (ALDH) enzymes, is linked to radiation resistance and tumor recurrence [17,18]. In malignancy, ALDH enzyme expression is not fixed in a specific cell type but fluctuates according to the disease state, stromal niche, and other factors, and is most likely involved in mediating metabolic adaptation and at least part of the CSCs’ resistance to drug-induced oxidative stress [17,19,20,21,22,23]. ALDH expression in hybrid EMT-like tumor stages has been reported, with example CSCs from ovarian clear cell carcinoma [24].

## 3. Maintaining and Keeping of Hematopoietic Stem Cells (HSCs) in the BM

**Figure 1:**
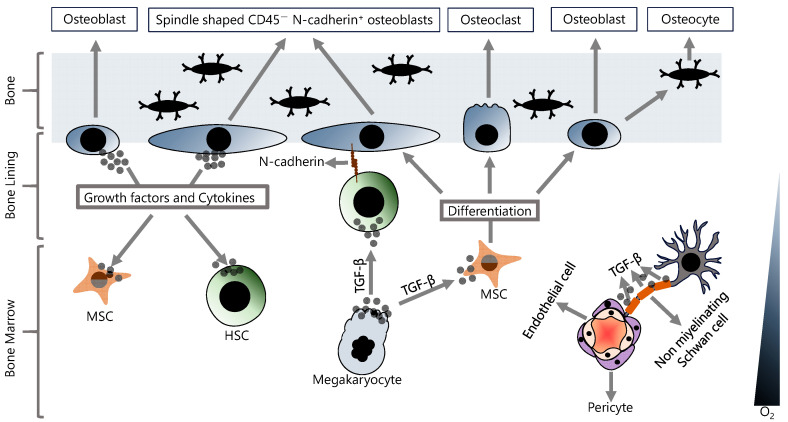
Hematopoietic and non-hematopoietic cells in the BM. In the BM, stem cell properties and differentiation signals are regulated by various cellular and non-cellular components, and their fine-tuned interactions are critical for maintaining homeostasis.

All blood cells, including immune system cells, develop from HSCs in the BM [25]. The BM is a highly complex environment composed of many hematopoietic and non-hematopoietic cells and non-cellular components (Figure 1) [26]. The HSCs are not randomly distributed in the BM and reside in specific microenvironments called niches [27]. BM niches contain a highly complex set of non-cellular factors, including various cytokines and growth factors, that are critical for regulating the functions of HSCs in the niche. Although multiple non-cellular factors have been implicated in the regulation of HSCs in the BM niche, the TGF-β family of signaling molecules plays a special role in this regulation. TGF-β is abundant in the BM milieu and is mainly produced and secreted by both hematopoietic cells and various BM niche cells such as megakaryocytes and non-myelinating Schwann cells [28,29,30]. TGF-β has been shown to promote HSC quiescence in BM and to strongly inhibit HSC growth. Neutralization of TGF-β by monoclonal antibody results in an increase in early progenitor cells from quiescent HSCs [31]. In addition, in vivo depletion of megakaryocytes was shown to promote differentiation and to inhibit quiescence in HSCs [32]. However, potential oncogenic functions of TGF-β have also been reported. For example, hypoxic BM microenvironment-dependent stimulation of TGF-β signaling induces CXCR4 expression and, thereby, promotes the survival of chemoresistant LSCs [33]. Consistent with this, it has been shown that inhibition of TGF-β and CXCR4 results in improved survival in an fms-related receptor tyrosine kinase 3 (*flt3*)-mutated acute myeloid leukemia (AML) model [33]. TGF-β in the BM milieu is also involved in the regulation of osteoblast differentiation from MSCs, but it should be noted that TGF-β signaling has opposite effects in the early and late stages of chondrogenic differentiation [34]. Indeed, TGF-β signaling is associated with multiple intracellular signaling mechanisms, including SMAD, MAPK, and AKT, and TGF-β-regulated chondrogenesis in BM is associated with crosstalk between MAPK, Wnt, and N-cadherin signaling [35]. Furthermore, the inhibition of BMP signaling by the deletion of the gene that encodes bone morphogenetic protein receptor type 1A (*bmpr1a*) in HSCs and stromal cells increased N-cadherin expression in both osteoblasts and HSCs [36]. The interactions between HSCs and various cellular components of the niche regulate the characteristics of HSCs, including self-renewal and quiescence, which are crucial for maintaining and sustaining the HSC pool [37], and these interactions are mainly provided by adhesion molecules [38,39].

At least two HSC niches have been identified in BM, an endosteal niche (also known as osteogenic niche) and a perivascular niche (also known as central niche) [40]. The vascular niche has a very rich vascularization consisting of arterioles, and the cells directly associated with the vasculature can be summarized as endothelial cells, perivascular stromal cells, peri-arteriolar Ng2^+^ cells, non-myelinating Schwann cells, megakaryocytes, and HSCs [41]. The endosteal niche has a hypoxic environment and this low-oxygen milieu allows HSCs to remain in a quiescent state, thus maintaining the stem cell pool [42]. The endosteal niche is physically close to the trabecular bone and contains many mesenchymal stem cells (MSCs) and cells of the osteoblastic lineage, including osteoblasts, osteocytes, and osteoclasts [43]. The MSCs are the multipotent stem cells with the ability to both self-renew and differentiate. Osteoblast cells differentiate from MSCs and play an important role in bone development [44]. Osteoblasts in the endosteal niche have been shown to express many cytokines and growth factors to support the stem cell population in the BM [45]. In fact, the osteoblast cell population in the endosteal niche is not uniform, and there are at least two subpopulations of osteoblasts; (1) osteoblasts that function in bone formation and (2) spindle-shaped CD45^−^ osteoblasts that express N-cadherin [46,47]. Overall, osteoblasts play a critical role in establishing and maintaining an essential niche microenvironment and are also involved in regulating stem cell quiescence and proliferation, in addition to bone formation.

HSCs in the BM niche are under both endogenous and exogenous stress conditions, and these stress factors can lead to DNA damage that may result in mutations if not properly repaired, and consequently, the accumulation of mutations may result in malignant transformation [48]. The malignantly transformed HSCs are referred to as leukemic stem cells (LSCs), and the gene expression profile of these cells, in addition to their mutational status, is very different from that of HSCs [49]. In addition, it has been reported that leukemia cells also secrete TGF-β, and autocrine TGF-β signaling may lead to phenotypic variation, which may be the main cause of leukemia cell heterogeneity [50]. Although LSCs have self-renewal and differentiation abilities like HSCs, these cells are abnormal and have the capacity to initiate leukemia; therefore, they are also called leukemia-initiating cells (LICs) [51,52]. Leukemias are a heterogeneous class of hematologic malignancies that result from the proliferation of immature and non-functional leukocytes, called blasts, in the BM and are classified as acute or chronic leukemias depending on the proportions of these abnormal leukocytes in the BM [53,54]. The malignant transformation of HSCs into LSCs changes the BM niche environment into a new milieu that supports leukemogenesis [55]. These changes are at least partially associated with signals from leukemic cells, and these events consequently further support leukemic cells [56,57]. In fact, changes in the structure and nature of the niche contribute to the creation of an environment that does not support normal hematopoiesis but contributes to disease progression [58]. The altered niche environment induces further damage to HSCs, transforming them into pre-leukemic and leukemic cells. Furthermore, it has been shown that the dysfunction of osteolineage cells in the BM niche can induce myelodysplasia and leukemia [57]. Consequently, the LSC-induced microenvironment creates novel environmental conditions that protect leukemia cells from chemotherapy [59]. For example, classical chemotherapeutic agents that cause DNA damage or spindle poisoning target actively proliferating cells and therefore cannot be used effectively against quiescent LSCs in the BM niche [60]. The interactions between LSCs and BM niches are important for leukemic cells, and these associations may control many vital mechanisms, including the promotion of survival, inhibition of apoptosis, and resistance to chemotherapy [61].

## 4. E-Cadherin and N-Cadherin, the Main EMT Markers, Have Crucial Roles in BM Homeostasis and Hematopoiesis, and Also in Leukemogenesis

EMT is an important cellular process characterized by the impairment of cell–cell adhesion properties and is associated with poor prognosis in cancer [62]. It is generally characterized by a decrease in E-cadherin expression and an increase in N-cadherin expression [63]. Although cadherin molecules are normally involved in cell–cell connections, they also have important roles in the interaction between cancer cells and the tumor microenvironment in solid tumors. In the context of hematologic malignancies, cadherin proteins are important, like solid tumors, and play a role in the interaction between leukemic cells and BM stromal cells.

### 4.1. E-Cadherin in Acute Leukemia

E-cadherin is a member of the Ca^2+^-dependent cadherin protein family, and mature E-cadherin contains three distinct domains, which are the highly conserved carboxy-terminal cytoplasmic domain, a single-pass transmembrane domain, and an N-terminal extracellular domain [64]. The N-terminal extracellular domain has five extracellular cadherin repeat subdomains to form trans-cadherin interactions between neighboring cells, and is therefore essential for homophilic calcium-dependent cell–cell adhesion [65]. The correct conformational organization of cadherin extracellular domains is controlled and regulated by the binding of EC subdomains to Ca^2+^ [66]. Cadherin extracellular domains associate with the cytoplasmic tail to form signaling hubs called adherens junctions (AJs) [67]. The carboxy-terminal cytoplasmic domain of E-cadherin is required for the formation of cadherin–catenin complexes [68]. The full cadherin–catenin complexes are crucial for the adhesion of cells together to maintain epithelial tissue formation and stability [69]. In fact, one of the most important functions of the cytoplasmic region of E-cadherin is to bind to catenins, which link the cadherin to the actin cytoskeleton. This region has a catenin-binding domain (CBD) and a juxtamembrane domain (JMD) that bind β-catenin and p120-catenin, respectively [70]. CBD-bound β-catenin binds to α-catenin to form complete cadherin–catenin complexes [70]. On the other hand, E-cadherin found in epithelial cells also interacts with some surface markers found on some subsets of T cells to modulate the immune system [71]. E-cadherin has been shown to interact with CD103 found on cytotoxic T lymphocytes (CTLs) and tissue-resident T lymphocytes (TREMs), activating the cytotoxic functions of these cells in a TCR-dependent manner [72,73]. E-cadherin also interacts with killer cell lectin-like receptor G1 (KLRG1) on the surface of NK and CD8+ cells, inhibiting TCR signaling and thus the effector functions of these cells [74,75,76].

E-cadherin was shown to be involved in the differentiation and maintenance of stem cells. In this context, E-cadherin is reported to be overexpressed in embryonic stem cells (ESCs) and is required for the pluripotency of ESCs and their differentiation [77]. The link between E-cadherin and hematopoiesis has been demonstrated in erythroid progenitors and it has been shown that E-cadherin has an erythroid lineage-restricted expression in BM cells and is involved in the maturation of erythroid progenitors [78]. Consistent with this, it was shown that E-cadherin is predominantly expressed in the basophil/mast cell lineage in mouse BM and that inhibition of E-cadherin causes a disruption of erythroid differentiation [78,79,80]. However, E-cadherin does not seem to be expressed in human basophils [79]. Although it is not easy to explain this phenomenon based on the existing literature, it can be envisioned as being a result of the evolutionary process.

E-cadherin expression has been shown to be restricted to the erythroid lineage and may be expressed in AML blasts only when erythroid differentiation has occurred [78]. However, recent studies have shown that E-cadherin expression is decreased in acute leukemias, similar to solid tumors, and this event can be regulated by genetic and epigenetic mechanisms [81]. Indeed, the *cdh1* mRNA level has been shown to be significantly decreased in AML patients compared to healthy controls [82]. The patients with low *cdh1* expression and normal cytogenetics have shown a shorter overall survival, and therefore it has been suggested that the *cdh1* expression level can be used as an independent prognostic factor for AML [82]. Suppression of *cdh1* expression by promoter methylation has been demonstrated in various cancers including prostate and breast, and was reported to be associated with higher-grade cancers [83]. Consistently, E-cadherin expression was found to be suppressed by methylation of the 5′ CpG island of the *cdh1* promoter in acute leukemia [84,85]. Similar to solid tumors, promoter methylation-mediated silencing of *cdh1* has also been shown to be associated with poor prognosis in AML [86]. MALAT-1, an lncRNA, was shown to interact with enhancer of zeste 2 polycomb repressive complex 2 subunit (EZH-2) and SUZ-12, the subunits of the polycomb repressive complex 2 (PRC2), which are directly involved in methylation of the *cdh1* promoter and thus suppress *cdh1* expression in AML [87]. In fact, MALAT-1-dependent silencing of *cdh1* expression is not unique to AML and has been shown in renal cell carcinoma, bladder cancer, and castration-resistant prostate cancer [88,89,90]. miR-149-3p was shown to inhibit EMT in U937 AML cells. Consequently, decreased miR-149-3p expression results in an increase in proliferative, migratory, and invasive behavior of these cells [91,92]. Consistently, S100 calcium binding protein A4 (*s100a4*) was shown to be overexpressed in AML, which can be due to decreased miR-149-3p expression [93]. Although the mechanism of miR-149-3p-mediated inhibition of EMT in leukemias has not yet been demonstrated, several target mRNAs were shown to be involved in miR-149-3p-mediated inhibition of EMT in various cancers. miR-149-3p binds to S100A4 mRNA and reduces its levels, thereby inhibiting S100A4-induced EMT in bladder cancer cells [94]. Furthermore, miR-149-3p was shown to inhibit EMT by targeting TIMP metallopeptidase inhibitor 2 (TIMP2) and cyclin-dependent kinase inhibitor 1A (CDKN1A) mRNAs in ovarian cancer [95].

### 4.2. N-Cadherin in Acute Leukemia

N-cadherin is a member of the classical cadherin family, like E-cadherin, and has similar localization and organization to E-cadherin, although they generally have opposite functions [96]. Normal epithelial cells do not express N-cadherin (or express it at low levels), but most advanced cancers derived from epithelial tissue were shown to have high levels of N-cadherin expression [97]. Although N-cadherin is not thought to have an oncogenic role in normal epithelial cells, its increased expression in epithelial cancers causes an increase in malignant behavior and leads to a more aggressive cancer cell phenotype [98]. In this regard, the association between increased N-cadherin expression and enhanced migratory and invasive abilities of epithelial-derived cancers has been demonstrated for many cancer types [64]. Consistently, increased N-cadherin expression was shown to be associated with an unfavorable prognosis in patients with epithelial-derived cancers [99]. The expression of N-cadherin is tightly regulated and it has a high expression in mesenchymal-derived cells and neural tissues [100,101]. N-cadherin is expressed in various cell types that reside in BM niches: these cells are osteoblasts and MSCs in the endosteal niche, and endothelial cells and pericytes in the perivascular niche [102]. Osteoblasts, which play a critical role in establishing and maintaining the required niche microenvironment, and in regulating stem cell quiescence and proliferation, express N-cadherin in the BM niche [59,103]. Although earlier reports suggested that loss of N-cadherin in osteoblasts or HSCs would not have a deleterious effect on hematopoiesis, recent studies have shown that osteoblast-dependent regulation of HSCs in the BM niche is directly related to homophilic N-cadherin interactions between osteoblasts and HSCs [104,105]. N-cadherin is involved in maintaining HSC quiescence, and N-cadherin deficiency was shown to disrupt HSC adhesion to the endosteum and consequently to inhibit long-term HSC engraftment [106,107]. Interactions between HSCs and osteoblasts are crucial for maintaining stem cell properties, and N-cadherin plays an important role in fulfilling this function [108]. Consistently, N-cadherin overexpression has been shown to increase the interaction between HSCs and the endosteal niche and to promote self-renewal and quiescence of HSCs [109]. In this context, there is important crosstalk between osteoblast cells and HSCs in the endosteal niche via cell membrane receptors, adhesion proteins, and secreted cytokines to regulate HSC quiescence, proliferation, and differentiation [103]. HSC–osteoblast interactions also facilitate HSC binding to other cells in the endosteal niche, and this event is important for HSC function [104]. Although the interactions between HSCs and osteoblasts can be mediated by many types of adhesion molecules, N-cadherin-mediated interactions have a key role in the behavior of HSCs. Indeed, N-cadherin-expressing osteoblasts were shown to support HSCs, and there is a positive correlation between the number of N-cadherin-positive osteoblasts and HSCs [36]. Furthermore, it has been suggested that N-cadherin-expressing osteoblasts may also support HSC expansion in response to BM radioablation [110]. On the other hand, N-cadherin has also been shown to be involved in the maintenance of pre-osteoblast cells in the endosteal niche and, thus, has an important function in the regulation of osteogenesis [111].

The implication of N-cadherin in the interaction between cancer cells and the surrounding tumor microenvironment has been made in various solid tumors [102], and a positive relationship has been demonstrated between increased N-cadherin expression and resistance to chemotherapeutic agents [112]. Consistent with this, silencing of N-cadherin has been shown to drive cells into apoptosis [102]. Although the mechanism of resistance of cells with high levels of N-cadherin to chemotherapy is not fully understood, it was shown that N-cadherin inhibits the activities of some pro-apoptotic proteins while increasing the activity of anti-apoptotic proteins, generally in an AKT-dependent manner [113]. Similar to solid tumors, N-cadherin expression is increased in many types of hematologic malignancies as well as in BM niche-primed leukemia cells [114,115,116], and N-cadherin-mediated adhesion supports the resistance of leukemia cells to chemotherapeutic agents [117]. Furthermore, a negative correlation between response to chemotherapy and N-cadherin expression in LSCs has been shown in AML patients [118]. Indeed, AML patients that have higher-N-cadherin-expressing LSCs are more resistant to chemotherapy [118]. In line with this observation, it has been shown that the use of an antagonist peptide that would disrupt N-cadherin interactions between BM and chronic myeloid leukemia (CML) cells increases the sensitivity to imatinib [117]. Similar results have also been recorded in a BCR-ABL ALL cell line, and it was reported that the use of the same N-cadherin antagonist peptide inhibits the adhesion of ALL cells to fibroblasts [119]. Mechanistically, leukemic cells have been shown to interact with BM cells via N-cadherin, and this event facilitates their survival and escape from apoptosis and, consequently, resistance to treatment [116]. Indeed, depletion of N-cadherin was shown to decrease the proliferation of leukemic cells and sensitize them to dexamethasone [116]. Interestingly, the presence of N-cadherin has also been shown to protect osteoblast cells from chemotherapy-induced catastrophic stress [120].

### 4.3. EMT-Inducing TFs in Acute Leukemia

EMT can be induced by several TFs, including zinc finger E-box-binding homeobox (ZEB)1/2, twist family bHLH transcription factor 1 (TWIST1), and snail family transcriptional repressor (SNAIL)1/2 [121]. In fact, although these TFs have crucial roles in embryogenesis and their deficiencies are associated with multiple lethal defects, the implication of these proteins in cancer progression is much more extensively discussed. In this context, the association between these TFs and cancer progression/therapy resistance is relatively well established [122]. Although this relationship is generally discussed in terms of solid tumors, these EMT-inducing factors also play critical roles in both normal hematopoiesis and hematological cancers, including leukemia [121,123].

ZEB1/2 are EMT-inducing TFs that bind directly to the promoters of genes critical for EMT and regulate their expression [124]. The best-studied targets of ZEB proteins are *cdh1* and *cdh2*, which encode E-cadherin and N-cadherin, respectively [125,126]. Although most of the papers on the implication of ZEB proteins in cancer generally focus on solid tumors, there are also many reports on the effects in acute leukemia [127,128]. Indeed, an elevated ZEB1 level was associated with a more aggressive phenotype and poor prognosis in AML [129,130]. Consistently, depletion of ZEB1 has been shown to decrease cell proliferation and invasion in various in vitro and in vivo AML models, as well as to delay tumor formation in xenograft models [129,130]. Although a differential expression of ZEB2 in AML samples compared to healthy counterparts has not been reported, an association between ZEB2 levels and an EMT-like gene expression signature has been reported [131]. Consistent with this, depletion of ZEB2 was shown to decrease cell proliferation in both in vitro and in vivo AML models [9,10]. Indeed, ZEB2 has been shown to be involved in the differentiation and proliferation of AML cells and is also a driver of early thymic progenitor T-cell acute lymphoblastic leukemia (T-ALL) [127,132]. There is a negative association between miR-200 family members and *Zeb1/2* expression, and it was shown that miR-200 family members suppress *Zeb1/2* expression and consequently inhibit EMT [133]. Conversely, ZEB1/2 also inhibit the transcription of these miRNA family members and induce EMT [134]. In the context of leukemia, the expression of at least some members of the miR-200 family has been shown to be decreased in both AML and ALL [135,136].

TWIST1 is involved in the hematopoiesis and promotes the self-renewal of the CSCs, and, therefore, its expression is high in hematopoietic cells and LSCs, as expected [137]. Although TWIST2 has a high similarity to TWIST1 based on their primary sequences, it has a different expression pattern and function in both normal development and cancer progression. In this context, TWIST1 is involved in the development of the myeloid lineage, whereas TWIST2 inhibits myeloid cell development and increases the population of mature myeloid cells [138]. In the context of EMT, TWIST1 binds directly to the *cdh1* promoter, recruits methyltransferases, and, thereby, represses *cdh1* expression [139]. It also binds to the first intron of *cdh2* and promotes its expression [140]. Consequently, decreased (or lost) E-cadherin expression and increased N-cadherin expression due to increased TWIST1 activity drive cells into EMT [141]. This is the classic cadherin-switching mechanism and is generally associated with an unfavorable prognosis in solid tumors [142]. Although the role of TWIST1 in promoting both aggressiveness and therapy resistance is relatively well documented in solid tumors, it also plays a critical role in the pathogenesis of leukemia and other hematological malignancies. TWIST1 expression is high in AML cells, and its elevated level promotes cell proliferation and colony formation [143,144]. Consistently, TWIST1 levels were also observed to be high in acute promyelocytic leukemia (APL) cells, a sub-population of AML, and this event was shown to be due to its interaction with tribbles pseudokinase 3 (TRIB3), protecting it from degradation processes [145]. In addition, elevated TWIST1 expression was associated with resistance to apoptosis in AML [144] and with resistance to all-trans retinoic acid (ATRA) therapy in APL [145]. Moreover, TWIST1 was shown to promote stemness by inducing the expression of BMI-1, which is an important factor in the self-renewal of CSCs [146,147]. BMI-1 boosts the malignant behavior of CSCs by promoting EMT [148]. Indeed, miR-218 targeting BMI-1 was shown to inhibit proliferation in APL [149].

SNAIL1 has been shown to interact with and recruit HDAC1/HDAC2 and SIN3A to the *cdh1* promoter, and accordingly, SNAIL1 level is associated with histone 3 and histone 4 de-acetylation at the *cdh1* promoter [150]. Although this mechanism has not yet been demonstrated in acute leukemia cells, we can assume that this is a general mechanism for SNAIL1-mediated inhibition of E-cadherin expression. It was shown that the SNAIL1 level is higher in AML patients compared to controls [128]. Indeed, increased SNAIL1 expression was associated with impaired differentiation in AML cells, promoting self-renewal of CSCs and proliferation of immature myeloid cells, and this malignancy-promoting effect of SNAIL1 is dependent on the interaction with LSD1, a histone de-methylase [151]. In an animal model, SNAIL1 overexpression has been shown to lead to leukemia formation, and it also contributed to the development of radiotherapy resistance [152]. SNAIL2 has been shown to be overexpressed during leukemogenesis, and an increased SNAIL2 level contributes to apoptosis resistance in LSCs [153,154]. In line with this, it was shown that SNAIL2 downregulated the expression of p53-upregulated modulator of apoptosis (*puma*) in an ERK-dependent manner and, thereby, contributed to cytarabine resistance in leukemia cells [155]. miR-200 family miRNAs have EMT-inhibiting roles as described above, and a reciprocal inhibitory loop operates between miR-200 family members and SNAIL2: increased expression of the miR-200 family members inhibits EMT via suppression of SNAIL2 [156]. Another similar feedback mechanism exists between miR-203, an miRNA that is downregulated in AML, and SNAIL1: decreased miR-203 expression drives cells into EMT [157,158].

## 5. β-Catenin in Acute Leukemia

β-catenin plays an important role in both the establishment and stability of adherens junctions by binding to cadherin proteins, thus contributing to cell–cell junctions, and increased β-catenin activity has been shown to induce EMT [159]. β-catenin is a multifunctional protein localized to the nucleus, cytosol, and centrosomes in addition to adherens junctions [160], and its levels are tightly regulated mainly by GSK3β-dependent phosphorylation and subsequent destruction by proteasomal degradation mechanisms [161]. In fact, the cytosolic β-catenin level is normally low in cells because excess β-catenin is rapidly targeted by the proteasome in a ubiquitin-dependent manner [162]. However, inhibition of GSK3β leads to accumulation of β-catenin in cells and, thus, to its activation [161]. In addition, the cellular level and the activity of β-catenin may also be regulated by Wnt-independent mechanisms including PI3K/AKT [163]. β-catenin activation can be defined as the interaction of β-catenin with co-regulators in the nucleus, which is followed by its binding to the promoters of target genes together with the co-regulators, and consequently, the controlling of the transcription of these genes.

**Figure 2:**
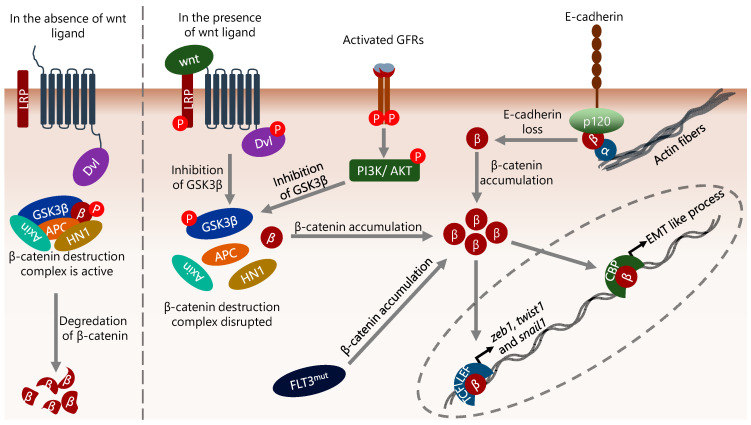
The diversity of mechanisms that influence β-catenin activity. β-catenin is important in the induction of EMT, and its level is mainly regulated by proteasomal degradation mechanisms in a GSK3β complex-dependent manner. Disruption of the GSK3β complex by Wnt or growth factor receptor (GFR), *flt3* mutation (internal tandem duplication), or reduction/loss of E-cadherin results in cytoplasmic accumulation of β-catenin. Cytoplasmic β-catenin translocates to the nucleus, binds directly to the promoter of target genes, and consequently regulates their expression. In the context of EMT, the genes encoding EMT-inducing TFs such as *zeb1*, *twist1* and *snail1* are targets of β-catenin. In addition, β-catenin binds to the promoter of genes that induce EMT-like processes in cells and increases their transcription. α, β, and p120 represent α-catenin, β-catenin, and p120-catenin, respectively.

In this context, nuclear β-catenin has been shown to interact with CREB-binding protein/E1A-binding protein p300 (CBP/p300) transcriptional co-activators and other basal transcriptional machinery apparatus member proteins [164]. In addition, β-catenin can bind to the nuclear TFs TCF/LEF and mediate the transcription of genes involved in cell proliferation such as *ccnd1* (encodes cyclin D1) and *Myc* [165]. Aberrant β-catenin activity can result from a variety of mechanisms, including epigenetic alterations, defects in upstream activating signals such as Wnt and AKT, activating mutations in β-catenin, mutations in the GSK3β/APC/Axin complex, increased hematological and neurological expressed 1 (HN1) level and the interaction status of β-catenin with cadherins [166,167,168,169] (Figure 2). In this context, although the status of E-cadherin and N-cadherin is generally considered in terms of the migratory abilities of epithelial cells by relating to adherens junctions, they also interact with β-catenin in the cytoplasm, and this interaction contributes to the regulation of both the cellular level and intracellular localization of β-catenin [69]. In addition, various cell-specific defects can also induce abnormal β-catenin activity, such as *flt3* mutations in AML. *flt3* internal tandem duplication is a common defect in AML and is associated with poor prognosis [170,171]. This mutation was shown to increase the β-catenin level, thereby promoting TCF/LEF-dependent transcription [172] (Figure 2).

Aberrant activation of β-catenin in HSCs has been shown to lead to cell cycle entry and subsequent exhaustion of HSCs in BM [173]. In this context, the co-activators that interact with β-catenin are important for stem cell functions, and CBP and p300 were shown to be involved in self-renewal and differentiation of HSCs, respectively [174]. In fact, the effects of CBP and p300 in this way are not restricted to HSCs, and they act in a similar way in ESCs [175]. However, although CBP and p300 have been identified as bimodal regulators of β-catenin signaling and transcriptional activity, it is unclear to what extent β-catenin interactions with CBP or p300 affect cell fate, i.e., self-renewal or differentiation [176]. It has also been shown that β-catenin is associated with CBP in the nuclei of cells undergoing an EMT-like process [177]. However, it appears that the effect of β-catenin in inducing EMT is not limited to interaction with CBP. For example, the β-catenin/TCF4 complex has been shown to bind directly to the *zeb1* promoter, increasing its expression and thereby inducing EMT [178,179]. On the other hand, it was also shown that EMT-promoting TFs can induce β-catenin expression. In this context, miR-200a, which represses *zeb1/2* and *snail2* expression and consequently inhibits EMT, also inhibits *β*-*catenin* expression in a ZEB1/2-dependent manner [180]. β-catenin can also bind to the promoters of genes encoding other EMT-inducing TFs, such as *snail1* and *twist1*, in a complex with TCF/LEF family proteins, and regulate their transcription [181]. It has also been shown that β-catenin, which has a mutation that allows it to localize to the nucleus, causes a decrease in the levels of some cell–cell junction molecules, including E-cadherin, and consequently induces EMT in colon cancer cells [182]. Although both studies were performed in non-hematologic cells, their results may represent a general mechanism for β-catenin-induced EMT, including HSCs and LSCs. The effect of β-catenin on HSCs appears to be dose-dependent. In this context, it has been reported that mildly activated β-catenin can increase the clonogenicity and myeloid development of HSCs, whereas highly increased activity causes a disruption of HSC functions such as self-renewal and differentiation [183,184]. Increased β-catenin levels have been demonstrated in many cancers [164], and there are numerous reports that aberrant activation of β-catenin is involved in the pathogenesis of many hematologic malignancies, including leukemia [185]. It has been shown that β-catenin is also involved in the transformation of healthy HSCs into LSCs [186]. Although the mechanism of transformation of HSCs into LSCs is not fully understood, the critical role of β-catenin and related genes in the pathogenesis of hematological malignancies has long been observed and discussed [185].

β-catenin has been shown to be required for the development of AML and ALL LSCs [187,188], and an activating mutation in β-catenin alters the differentiation potential of myeloid progenitors and consequently causes AML development [189]. Consistently, the β-catenin level is significantly increased in AML cells, accompanied by a decrease in the E-cadherin level, and this event has been associated with poor prognosis [190,191,192,193]. Furthermore, the level of β-catenin has been shown to be higher in samples from relapsed AML patients and in BM-resident leukemic cells compared to samples from circulating blasts [194]. Accordingly, disruption of β-catenin signaling has been shown to have a potent effect against AML CSCs and also to have a synergistic effect with FLT3 inhibition on the *flt3*-mutant AML cells [194]. However, it has also been reported that genetic deletion of β-catenin in LSCs does not affect their ability to self-renew, contrary to previous reports [195]. To this point, it should be noted that although many independent studies have shown the strong implication of β-catenin in the transformation of HSCs, and thereby the development of LSCs, the aberrant expression or activation of β-catenin as a single factor is not sufficient for the development of leukemia [187,196,197]. On the other hand, moderate β-catenin activity in the stromal cells is also required for a balanced microenvironment that supports healthy hematopoiesis in the BM. In this context, β-catenin depletion in BM stromal cells has been shown to cause a decrease in HSC maintenance, whereas increased β-catenin results in an enhancement of HSC functions such as self-renewal [198,199]. Similarly, constitutively active β-catenin in osteoblasts residing in the BM niche impairs hematopoiesis and drives leukemogenesis [189]. Consistent with this, it has been shown that more than 35% of AML patients have increased β-catenin activity in osteoblasts in the BM [189].

## 6. Conclusions

**Figure 3:**
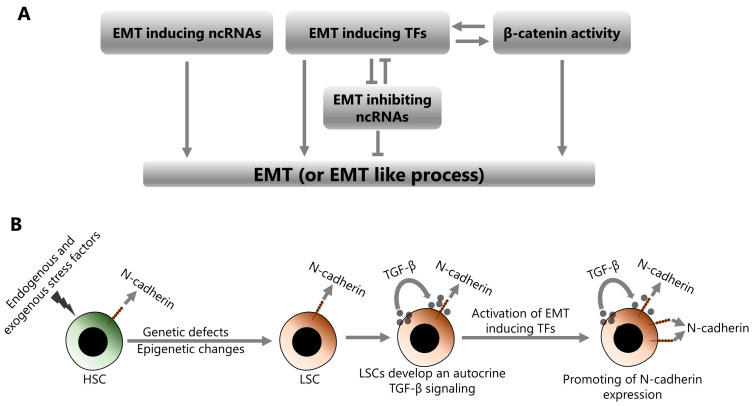
Induction of EMT or EMT-like process in leukemic cells. (**A**) EMT can be induced by the increased expression of EMT-inducing ncRNAs, increased activities of EMT-inducing TFs and increased activity of β-catenin. EMT-inducing TFs and β-catenin may enhance each other’s EMT-inducing activities. EMT-inducing TFs may also repress the expression of EMT-inhibiting ncRNAs. (**B**) The transformation of HSCs into LSCs, the stepwise induction of EMT in leukemic cells, and the increase in N-cadherin expression.

EMT is a dynamic cellular process that gives the cells the ability to adapt to new functions. This process is crucial during embryogenesis but is also involved in increasing the malignant behavior of cancer cells, especially in advanced stages, affecting properties that include stemness, proliferation, migration, invasion, and resistance to chemotherapy. EMT can be induced through various mechanisms, including epigenetics, EMT-inducing TFs, and β-catenin. Although EMT has been studied relatively extensively in solid tumors, it has also attracted attention in hematologic malignancies, especially in recent years. Acute leukemias represent a significant proportion of hematologic malignancies that affect both adults and children, and it has been shown that EMT is strongly involved in the pathogenesis of acute leukemias [200]. In this context, the current literature suggests a plausible model for the transformation of HSCs into LSCs and the induction of EMT in the transformed LSCs (Figure 3).

HSCs transform into LSCs by undergoing various genetic defects and epigenetic changes depending on the endogenous and exogenous stress factors to which they are exposed. Transformed LSCs establish an autocrine TGF-β signaling loop by both secreting TGF-β and stimulating themselves through the TGF-β they secrete. TGF-β signaling is one of the key mechanisms that stimulate the activation of EMT-inducing TFs such as ZEB1/2, TWIST1, and SNAIL1/2. Therefore, the activities of these molecules are increased in LSCs, depending on the elevated TGF-β signaling. The activities of EMT-inducing TFs are also promoted by various genetic and epigenetic factors in LSCs, including β-catenin over-activation. Consequently, increased activities of EMT-inducing TFs cause an increase in the N-cadherin expression. N-cadherin is an important adhesion molecule that is directly involved in the interactions between leukemic cells and the cellular components of the BM. The interactions between leukemic and stromal cells through N-cadherin promote the survival and dormancy of leukemic cells in the BM niche. Therefore, increased N-cadherin expression promotes stemness in leukemic cells and also protects them from chemotherapy and radiotherapy. Overall, EMT and the resulting high level of N-cadherin, in leukemic cells, is associated with increased malignant behavior, treatment resistance, and also poor prognosis in patients with acute leukemia. Indeed, a recent paper has shown that EMT-related gene expression signatures may be useful in predicting the prognosis of AML patients [201]. Therefore, monitoring EMT expression signatures to predict the prognosis of acute leukemia patients seems to be a practical approach. Moreover, comprehensive targeting of the EMT process, in addition to conventional therapies, is likely to improve the treatment success in these patients. However, the applicability of this strategy may not be realistic, due to both the technical and biological difficulties of targeting a whole mechanism and its possible unpredictable consequences. To this end, specific targeting of N-cadherin seems to be more practical. Indeed, the robust association between high N-cadherin expression and poor prognosis in patients with acute leukemia makes it an important therapeutic target. In concordance, ADH-1, which is an N-cadherin antagonist peptide with FDA approval for the treatment of solid tumors, has been shown to both inhibit the malignant behavior of ALL cells and increase leukemia cell death by apoptosis [116].

On the other hand, the critical relationship between the EMT process and poor prognosis/therapy resistance requires further investigation of this process in acute leukemias. In this context, bioinformatics and artificial intelligence approaches can also be used to identify EMT-related molecules that can be used both as prognostic biomarkers and/or therapeutic targets. Indeed, a recent paper has demonstrated SMAD7 and SERPINE1 as the “tipping points of TGF-β induced EMT” using a computational approach [202].

## Data Availability

Not applicable.
